# Analysis of patient flows for orthopedic procedures using small area analysis in Switzerland

**DOI:** 10.1186/1472-6963-6-119

**Published:** 2006-09-15

**Authors:** Klazien Matter-Walstra, Marcel Widmer, André Busato

**Affiliations:** 1 Institute for Evaluative Research in Orthopedic Surgery, MEM centre, University of Bern, Stauffacherstrasse, Bern, Switzerland

## Abstract

**Background:**

In general cantons regulate and control the Swiss health service system; patient flows within and between cantons are thereby partially disregarded. This paper develops an alternative spatial model, based upon the construction of orthopedic hospital service areas (HSA_O_s), and introduces indices for the analysis of patient streams in order to identify areas, irrespective of canton, with diverse characteristics, importance, needs, or demands.

**Methods:**

HSA_O_s were constructed using orthopedic discharge data. Patient streams between the HSA_O_s were analysed by calculating three indices: the localization index (% local residents discharged locally), the netindex (the ratio of discharges of nonlocal incoming residents to outgoing local residents), and the market share index (% of local resident discharges of all discharges in local hospitals).

**Results:**

The 85 orthopedic HSA_O_s show a median localization index of 60.8%, a market share index of 75.1%, and 30% of HSA_O_s have a positive netindex. Insurance class of bed, admission type, and patient age are partially but significantly associated with those indicators. A trend to more centrally provided health services can be observed not only in large urban HSA_O_s such as Geneva, Bern, Basel, and Zurich, but also in HSA_O_s in mountain sport areas such as Sion, Davos, or St.Moritz. Furthermore, elderly and emergency patients are more frequently treated locally than younger people or those having elective procedures.

**Conclusion:**

The division of Switzerland into HSA_O_s provides an alternative spatial model for analysing and describing patient streams for health service utilization. Because this small area model allows more in-depth analysis of patient streams both within and between cantons, it may improve support and planning of resource allocation of in-patient care in the Swiss healthcare system.

## Background

Since January 1997, all Swiss hospital discharges are collected yearly in the Swiss Federal Statistical Office's medical statistics of stationary institutions. Each discharge record is labelled with a residence code called medstat, which is an aggregate of several postal code areas. Each medstat region has 3'500 – 10'000 inhabitants and is created according to socio-economic and geographic coherence criteria. Switzerland is divided into 612 medstat regions, of which 240 contain at least one hospital [1]. After a start-up period, from 2000 on the data collection may be considered complete. By means of these data, an exact inventory of the status of the Swiss health care supply and hospital usage can be established. In addition to a traditional analysis based on cantons, studies based on hospital service areas (HSAs) can be performed. HSAs are aggregates of medstat regions in which at least one medstat region with at least one hospital is represented. Their definition is based on the small area analysis methodology described for health service research [2,3].

The segmentation of Switzerland into HSAs offers a meaningful spatial model that enables more detailed examination of stationary hospital services used by HSA residents and nonresidents. Affording insight into the geographical distribution of hospital usage [4-8], HSAs enable the description of variability in patient flows and measurement of the extent of local and nonlocal (to an HSA) treatments. Several indices that describe patient streams can help identify areas that attract and treat local or nonlocal residents, and HSAs allow more precise analysis of potential health supply shortages or overcapacities. Also, the focus of HSA studies can be sharpened to single medical disciplines (internal medicine, surgery, etc.), individual diagnoses (ICD10 [9]), specific treatments (CHOP-codes, a translation and adaptation of the US classification ICD-9-CM volume 3, [10]), one type of hospital (acute, rehabilitation), or applied to insurance-based accommodation type (private, semi-private, or general). Instead of using the previously defined general hospital service areas by Klauss et al, this paper defines orthopedic hospital service areas (HSA_O_s) that use Swiss orthopedic discharge data from 2000–2002. There were several reasons for defining orthopedic HSA. First the main focus of the research in our institute is on orthopedics. Secondary it is well known now that the federal discharge data from 1998 until 2000 were not as complete as the later data. The second data set ordered from the Swiss Federal Office of statistics obtained only orthopedic procedures for the years 2000–2002, but with much more patient information as the first data set. Third, because within the different time periods of the data sets, in which hospitals were closed, pooled together or newly opened based on a new health insurance law in Switzerland, it was decided to build new and for this study orthopedic specific HSAs.

The study describes patient flows within the country using a model based on small areas instead of larger administrative, cantonal areas. This focus on patient flows rather than on utilization was chosen as literature describing the use of small area analysis for analysing patient streams is hardly available. The study shows how certain partially newly defined patient stream indices can identify diverse, important characteristics of areas, and may assist in the formulation of hypotheses to explain variability in patient flows.

## Methods

### Definition of hospital service areas for orthopedic procedures

To construct hospital service areas for orthopedic procedures (HSA_O_s), a medstat of a person's residence (home medstat) and medstat where treatment took place (treatment medstat) must be available for each hospital discharge (as described in detail by Klauss [3] and Goodman[2]). Briefly, HSA_O _definition consists of 3 steps:

▪ For each home medstat region, the treatment medstats in which the first, second, and third highest number of discharges have taken place are calculated. For medstat regions with at least one hospital, home medstat and treatment medstat can be the same. Thereafter all medstat regions are allocated to the treatment medstat in which the highest number of discharges has taken place (primary HSA_O_). Theoretically for Switzerland, there now may be 240 HSA_O_s as Switzerland has 240 medstat regions with at least 1 hospital.

▪ The medstat regions are displayed in the Swiss medstat map (ArcMap) according to the assigned treatment medstat with the highest number of discharges. HSA_O_s are then examined for violations of the plurality rule [3,11]. This means that if the sum of discharges in the second- and third-place medstats is much higher than the one for the assigned medstat, and the second and third-place medstat belong to the same HSA_O _"X", the medstat in question is also assigned to this HSA_O _"X". In addition, if medstats are geographically disconnected from their primary HSA_O_, they also may be assigned to an adjacent HSA_O _if this HSA_O _contains the second- or third-place medstat (continuity).

▪ In the third step, discharge numbers are calculated on the basis of the HSA_O_s. If the highest number of discharges is not in the own HSA_O _the plurality rule demands that the HSA_O _should be allocated to the HSA_O _where the highest number of discharges take place. A final HSA_O _shape file is dissolved out of the aggregated medstats.

### Calculated indicators

A basic data set per HSA_O _contains 3 indicators that permit the calculation of further indices (numbers or ratios). The 3 basic indicators per HSA_O _are number of HSA_O _residents discharged (pop_d), number of residents discharged in home HSA_O _(local_d), and total number of discharges in HSA_O _hospital(s) (hosp_d). On the HSA_O _level the following indicators can be calculated:

▪ Localization index (LI): local_dpop_d∗100%
 MathType@MTEF@5@5@+=feaafiart1ev1aaatCvAUfKttLearuWrP9MDH5MBPbIqV92AaeXatLxBI9gBaebbnrfifHhDYfgasaacH8akY=wiFfYdH8Gipec8Eeeu0xXdbba9frFj0=OqFfea0dXdd9vqai=hGuQ8kuc9pgc9s8qqaq=dirpe0xb9q8qiLsFr0=vr0=vr0dc8meaabaqaciaacaGaaeqabaqabeGadaaakeaadaWcaaqaaiabdYgaSjabd+gaVjabdogaJjabdggaHjabdYgaSjabc+faFjabdsgaKbqaaiabdchaWjabd+gaVjabdchaWjabc+faFjabdsgaKbaacqGHxiIkcqaIXaqmcqaIWaamcqaIWaamcqGGLaqjaaa@4173@ : The localization index describes the percentage of HSA_O _residents who are discharged in their home HSA_O_.

▪ Netindex: (hosp_d−local_d)(pop_d−local_d)
 MathType@MTEF@5@5@+=feaafiart1ev1aaatCvAUfKttLearuWrP9MDH5MBPbIqV92AaeXatLxBI9gBaebbnrfifHhDYfgasaacH8akY=wiFfYdH8Gipec8Eeeu0xXdbba9frFj0=OqFfea0dXdd9vqai=hGuQ8kuc9pgc9s8qqaq=dirpe0xb9q8qiLsFr0=vr0=vr0dc8meaabaqaciaacaGaaeqabaqabeGadaaakeaadaWcaaqaaiabcIcaOiabdIgaOjabd+gaVjabdohaZjabdchaWjabc+faFjabdsgaKjabgkHiTiabdYgaSjabd+gaVjabdogaJjabdggaHjabdYgaSjabc+faFjabdsgaKjabcMcaPaqaaiabcIcaOiabdchaWjabd+gaVjabdchaWjabc+faFjabdsgaKjabgkHiTiabdYgaSjabd+gaVjabdogaJjabdggaHjabdYgaSjabc+faFjabdsgaKjabcMcaPaaaaaa@53AD@ or −1∗(pop_d−local_d)(hosp_d−local_d)
 MathType@MTEF@5@5@+=feaafiart1ev1aaatCvAUfKttLearuWrP9MDH5MBPbIqV92AaeXatLxBI9gBaebbnrfifHhDYfgasaacH8akY=wiFfYdH8Gipec8Eeeu0xXdbba9frFj0=OqFfea0dXdd9vqai=hGuQ8kuc9pgc9s8qqaq=dirpe0xb9q8qiLsFr0=vr0=vr0dc8meaabaqaciaacaGaaeqabaqabeGadaaakeaacqGHsislcqaIXaqmcqGHxiIkdaWcaaqaaiabcIcaOiabdchaWjabd+gaVjabdchaWjabc+faFjabdsgaKjabgkHiTiabdYgaSjabd+gaVjabdogaJjabdggaHjabdYgaSjabc+faFjabdsgaKjabcMcaPaqaaiabcIcaOiabdIgaOjabd+gaVjabdohaZjabdchaWjabc+faFjabdsgaKjabgkHiTiabdYgaSjabd+gaVjabdogaJjabdggaHjabdYgaSjabc+faFjabdsgaKjabcMcaPaaaaaa@5679@ when (hosp_d−local_d)(pop_d−local_d)
 MathType@MTEF@5@5@+=feaafiart1ev1aaatCvAUfKttLearuWrP9MDH5MBPbIqV92AaeXatLxBI9gBaebbnrfifHhDYfgasaacH8akY=wiFfYdH8Gipec8Eeeu0xXdbba9frFj0=OqFfea0dXdd9vqai=hGuQ8kuc9pgc9s8qqaq=dirpe0xb9q8qiLsFr0=vr0=vr0dc8meaabaqaciaacaGaaeqabaqabeGadaaakeaadaWcaaqaaiabcIcaOiabdIgaOjabd+gaVjabdohaZjabdchaWjabc+faFjabdsgaKjabgkHiTiabdYgaSjabd+gaVjabdogaJjabdggaHjabdYgaSjabc+faFjabdsgaKjabcMcaPaqaaiabcIcaOiabdchaWjabd+gaVjabdchaWjabc+faFjabdsgaKjabgkHiTiabdYgaSjabd+gaVjabdogaJjabdggaHjabdYgaSjabc+faFjabdsgaKjabcMcaPaaaaaa@53AD@ =< 1. The netindex describes the ratio of discharges of incoming nonlocal residents to discharges of outgoing local residents. High positive values mean an overall discharge influx of non-HSA_O _residents; a high negative value means an overall discharge outflow of HSA_O _residents into other HSA_O_s.

▪ On the HSA_O _hospital level the indicator "market share index"(MSI) [12] can be calculated: local_dhosp_d∗100%
 MathType@MTEF@5@5@+=feaafiart1ev1aaatCvAUfKttLearuWrP9MDH5MBPbIqV92AaeXatLxBI9gBaebbnrfifHhDYfgasaacH8akY=wiFfYdH8Gipec8Eeeu0xXdbba9frFj0=OqFfea0dXdd9vqai=hGuQ8kuc9pgc9s8qqaq=dirpe0xb9q8qiLsFr0=vr0=vr0dc8meaabaqaciaacaGaaeqabaqabeGadaaakeaadaWcaaqaaiabdYgaSjabd+gaVjabdogaJjabdggaHjabdYgaSjabc+faFjabdsgaKbqaaiabdIgaOjabd+gaVjabdohaZjabdchaWjabc+faFjabdsgaKbaacqGHxiIkcqaIXaqmcqaIWaamcqaIWaamcqGGLaqjaaa@42D2@ : it describes the percentage of HSA_O _resident discharges of all of the discharges (local and non-local HSA_O _residents) within the HSA_O_.

### Data

Federal discharge data for orthopedic procedures (according to CHOP and/or ICD10 codes) from the Swiss hospital discharge master file from 2000–2002 were used (Swiss Federal Office of statistics). Commercial GIS-compatible vector files for medstat regions were obtained from MicroGIS (MicroGIS Ltd, Baar, Switzerland).

The inclusion criteria for the total orthopedic dataset were as follow:

Primary or additional procedure CHOP codes 77.00–84.90. [10], and/or ICD10 primary diagnosis codes M00.0-M25.9, M40-M43.9, M45-M51.9, M53-M54.9, M60-M63.8, M65-M68.8, M70-M73.8, M75-M77.9, M75-M77.9, M79-M96.9, M99-M99.9 [9].

Additional variables in the dataset were age, admission type (emergency or planned procedures), and class of occupied bed. The class or accommodation type of the occupied bed is defined by the Swiss insurance system and means that a bed is covered by the respective insurance type. Both basic and semiprivate beds are provided in public hospitals, whereas private clinics only offer private insured beds. Insurance class of a bed does not necessarily relate to a patient's insurance coverage, for a patient may temporarily upgrade his/her insurance coverage to semiprivate for hospitalization in a semiprivate bed, which offers higher comfort with fewer beds per room (max two beds per room for semiprivate, one bed per room for private insured beds). The data set does not contain information on the insurance coverage of the patient; therefore the class of occupied bed is used as a surrogate marker to describe the effect of insurance coverage on the indices.

At the time the study was undertaken only data from 2000–2002 were available. As the Swiss federal institute for statistics is very restrictive in making data available newer data for this study (although possibly available by now) where not ordered later on. In addition, the Swiss healthcare system and hospital politics underwent larger re-organisations during the past 10 years, including hospital closures, pooling or new openings, and therefore data including larger time periods may distort results and lead to false conclusions.

### Statistical analysis

Statistical analyses where performed with SAS 9.1^® ^(SAS Institute Inc., Cary, NC, USA), geographic representations of data using ArcGis (ArcView8.2^®^, ESRI, Redlands CA, USA). Preliminary analyses indicated that most outcome variables were derived from symmetrical distributions; therefore univariate, linear models were used to analyse these data and the Bonferroni procedure was applied for pairwise comparisons in case of significant overall F tests. Residual analyses were applied to assess the fit between observed and modelled data, and R^2^-values were used to estimate the amount of variance of outcomes accounted for by the models. Correlations of continuous indicators with skewed distribution were assessed with Spearman's rho. The significance level was set at p < 0.05 throughout the study.

## Results

### Definition of HSA_O_s

HSA_O_s were built from 473,217 orthopedic discharges during the years 2000–2002. The allocation of medstat regions to their treatment medstat with the highest number of discharges (step 1) results in 115 primary HSA_O_s. 9% of the medstat regions were reallocated to a treatment medstat with second or third highest number of discharges (step 2) resulting in 85 HSA_O_s. The plurality check (step 3) required no further changes. The mean number of merged medstat regions per HSA_O _is 7.1 (95% confidence interval (CI) 1–21, Min = 1, Max = 41, Median = 5). 9.4% of the HSA_O _– four regions in Canton Graubünden, three in Canton Bern and one in Canton Appenzell Innerrhoden – enclose a single medstat. HSA_O_s containing 20–41 medstat regions (7%) correspond to the large urban regions Aargau, Bern, Geneva, Luzern, and Zurich. The average number of hospitals per HSA_O _is 4.35 (min 1, max 27), of which 21 (24.7%) have only 1 hospital.

### Variables

#### Localization index

**Figure 1:**
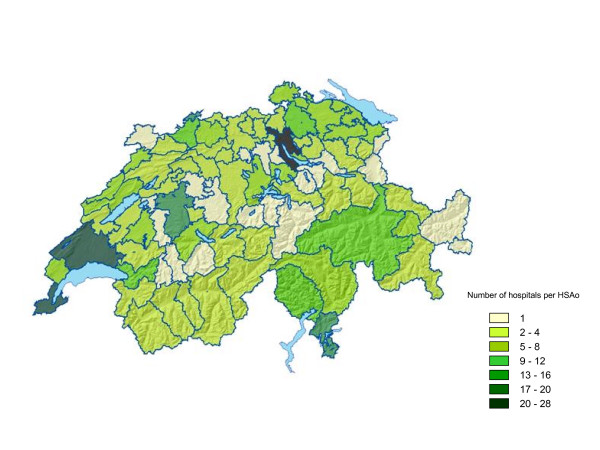
Number of hospitals per HSA_o_. Legend: Geographic distribution of the number of hospitals per HSA_o_.

**Figure 2:**
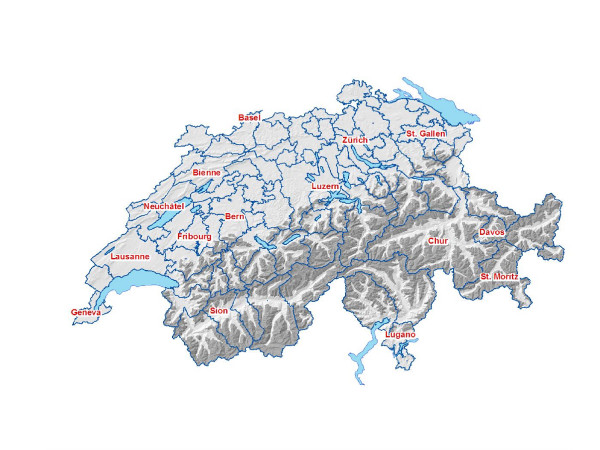
Geography of Switzerland, HSA_O _with major cities. Legend: blue lines = borders of HSA_o_, dark gray area = mountains.

**Figure 3:**
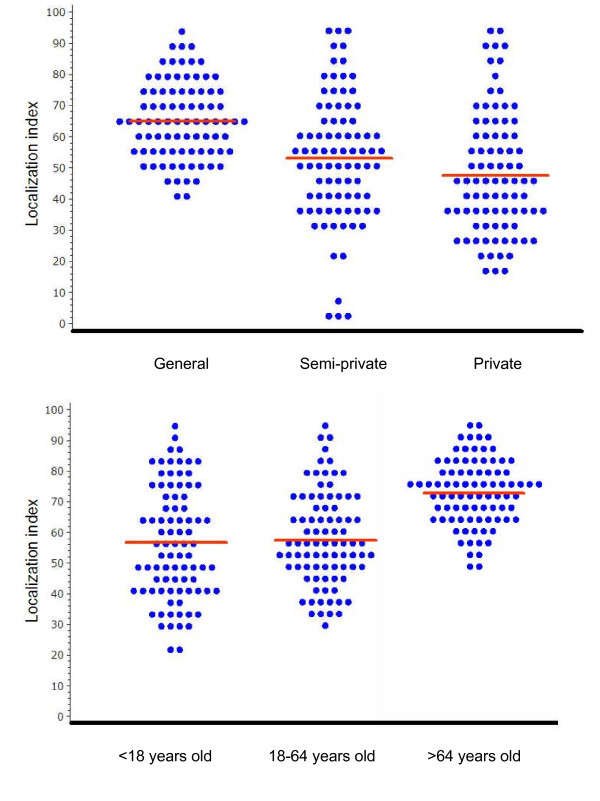
Localization index: class of occupied bed, age group. Legend: Turntip plot of the localization index per class of occupied bed and age group, ● = LI of single HAS_o _(Max. 25 in a row) – average.

The median localization index (LI, table 1, figures 2 and 3) for the total HSA_O _data over all 85 HSA_O_s is 60.8%. High LIs are mainly found in major large urban HSA_O_s such as Geneva (95.5%), Bern (90.8%), Lausanne (88.8%), Zurich (83.8%), Basel (83.4%), or the geographically more isolated Lugano (90.4%). Low LIs are found mostly for rural HSA_O_s. 20% of all HSA_O_s have a LI < 50%. The LI correlates significantly with the number of hospitals per HSA_O _(rho = 0.4613, p < 0.0001, figure 1).

The LI for all orthopedic data is significantly associated with insurance class of occupied bed (general, semiprivate, and private; table 1). The mean LI for general beds is 65.2% (median 64.5%), 52.9% for semiprivate beds (median 53.7%, p < 0.05 compared to general beds), and 47.9% for private beds (median 45.3%, p < 0.05 compared to general and semiprivate beds). Another factor associated with LI is age. LIs for children (< 18 years old) and adults (18–64) – respectively 56.5% (median 56.0%) and 57.8% (median 55.4%) – are significantly lower than the LI of 72.9% (median 73.7%) for seniors (> 64).

**Figure 4:**
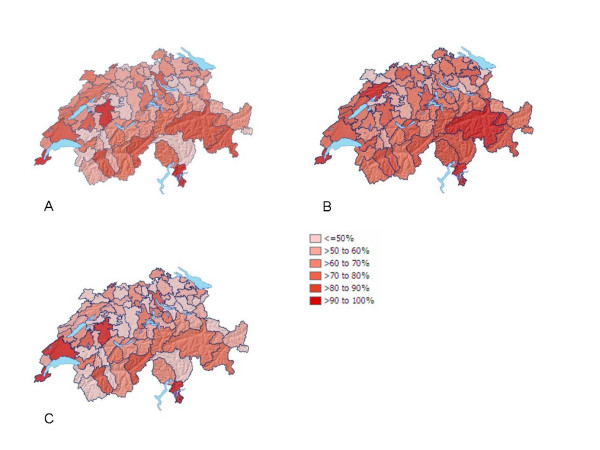
Localization Index for all, emergency and planned orthopedic procedures. Legend: Geographic distribution of the LI, A = all procedures, B = emergency procedures, C = planned procedures

For 6 HSA_O_s, including the large urban HSA_O_s Bern, Zurich, Aarau, and Fribourg, the localization index for private class beds is higher than for general beds. Another factor significantly associated with LI is the admission type (Figure 4). The average LI for emergency procedures, 72.0% (median 74.3%), is significantly higher than that for planned procedures: 57.3% (median 58.0%). Only six HSA_O_s show a LI < 50% for emergency procedures, while 29 HSA_O_s have an LI < 50% for planned procedures. Over the three years of the study, the average LI decreased by ± 1% per year (63.2% in 2000, 62.2% in 2001, and 61.1% in 2002, although the decrease was not significant). Residual analysis of statistical models indicated no violations of assumptions on the distribution of the underlying data, and models accounted for 14% of the variance of LI for insurance class, 20% for age group and 13% for admission type.

#### Netindex

The netindex is the ratio of HSA_O _resident discharges outside their home HSA_O_s to discharges of non-HSA_O _residents of other, surrounding HSA_O_s, resulting in the fact that the netindex for a single HSA_O _is not independent of values in neighbouring HSA_O_s. Therefore, only descriptive statistics for this index are given. Overall, 30.6% of HSA_O_s show a positive netindex, meaning that for one discharge outside a HSA_O _of residence, more than one discharge of nonlocal HSA_O _residents within that HSA_O _is registered (min -22, max 7.3).

Netindices between -2 and 2 are seen for 44% of the HSA_O_s. Elevated net inflows (netindex > 2) – with up to seven non-HSA_O _discharges coming in per one HSA_O _discharge going out – are seen for the large urban areas Bern, Zurich, and Lugano, as well as for the mountain areas Sion, Davos, and St.Moritz.

**Figure 5:**
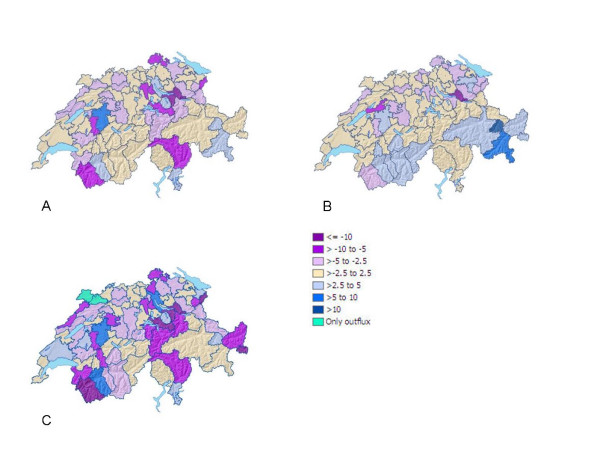
Netindex for all, emergency and planned orthopedic procedures. Legend: Geographic distribution of the netindex, A = all procedures, B = emergency procedures, C = planned procedures

High net outflows (netindex < -5) are observed in 11.7% of the HSA_O_, spread all over the country. The netindex correlates positively with the localization index (rho = 0.645, p < 0.001). The geographic distribution of the netindices is related to the admission type (Figure 5). Whereas high positive indices for emergency procedures (meaning a net influx of non-HSA_O _residents) are seen in HSA_O_s in mountain regions of the Bernese Alps (Saanen, Frutigen), Valais, and Graubünden, the distribution of net indices for planned procedures shows a different distribution, with a higher number of HSA_O_s with negative netindices (63 out of 85 (74.1%) vs. 46 out of 85 (54.1%) for emergency procedures).

Netindices are partially associated with the insurance class of the occupied bed (table 1). The percentage of HSA_O_s with a positive netindex is 31.8% for general beds, 23.2% for semiprivate beds, and 27% for private beds. Children (<18 years old) and adults (18–64 years old) show a lower percentage of HSA_O _with a positive netindex (29.4% and 25.9%) than seniors (>64 years old, 41.2%).

#### Market share index

**Figure 6:**
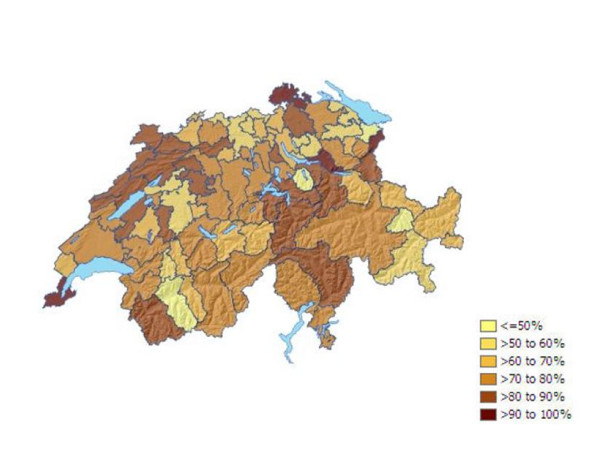
Market share index. Legend: Geographic distribution of the market share index

The mean market share index (MSI, table 1, figure 6) for all orthopedic data is 72.2% (median 75.1%). Four HSA_O_s (4.7%) – Sion, Davos, Einsiedeln, and Appenzell Ausserrhoden – have MSIs < 50%. Four other HSA_O_s – Geneva, Uznach, Schaffhausen, and Altstätten – have MSIs > 90%. The MSI correlates negatively with the netindex (rho = -0.715, p < 0.0001) but not with the LI (rho = 0.008).

The MSI is partially associated with the insurance class of occupied beds (table 1). For all orthopedic data, the difference between general beds (mean MSI = 73.3%) and semiprivate beds (mean MSI = 68.5%) is not significant. General and private beds (mean MSI = 65.7%) differ significantly (p = 0.0095), but between semiprivate and private beds there is no significant difference. For 18 HSA_O_s, including the large urban HSA_O_s Bern, Neuchatel, and Fribourg, the observed MSI for private class beds is higher than for general beds. The MSI for seniors, 76.8%, is significantly higher than those for the other two age groups. No significant differences of MSI were found between emergency and planned procedures.

### Interpretation

Localization index, netindex, and market share index all describe particular patient flows between HSA_O_s and HSA_O _hospitals, and may be interpreted in combination.

The localization index is an indicator of how frequently HSA_O _residents are treated within their own HSA_O_. The assumption that patients usually do not travel far in emergency situations is confirmed by a much higher average LI for orthopedic emergency treatments than for planned procedures. Furthermore, high localization and positive net indices for Geneva, Lausanne, Basel, Bern, Zurich, and Lugano indicate centralized health care utilization for emergency as well as planned procedures in the large urban areas. In contrast, typical rural HSA_O_s around those large urban areas show lower localization and negative net indices. This phenomenon may be explained by the fact that specialized orthopedic procedures are better or are only provided in large or specialized hospitals, whereas general procedures are obtainable almost everywhere.

LIs for specific patient groups may validate the hospital service areas. For example, Guagliardo et al. [13], using hospital areas defined by the Dartmouth Atlas of Healthcare, reported an up to 20% lower mean LI for paediatric hospitalisations. They concluded that children and seniors have significantly different geographic patterns of hospitalisation. For Swiss orthopedic data, the mean LIs for the age groups < 18 and 18–64 years old are also significantly lower than that for the group > 64. However, because the HSA_O_s are defined using all age groups (not using, as Guagliardo et al. did, a particular age group) the hospital service areas were assumed to be adequate for all ages.

Whereas no significant differences in LI, MSI, and netindex are observed between children and adults, as indicated by a higher average LI and MSI elderly patients seem in general to be served more locally than younger persons (< 65 years old) – an indication that elderly people tend to be less mobile than younger people.

The insurance class of occupied beds is clearly associated with the LI and netindex, with private beds showing the lowest average LI and highest negative net index. This may be due to the fact that patients demanding a private bed are more willing to travel outside their HSA_O_. When such a bed is not available in their own HSA_O_, they expect better treatment in another HSA_O_, or they seek treatment in a special clinic or demand specific treatments not provided in every hospital.

Netindices represent some typical Swiss geographic and socio-cultural settings. As a nation with large winter sport areas, HSA_O_s with a positive net index for emergency procedures are seen in the mountain areas in the Bernese Alps, Valais, and Graubünden. This may reflect the many leisure-related accidents involving non-HSA_O _residents that occur in these areas. In contrast, for planned procedures a positive net index is observed for the main large urban HSA_O_s of Lausanne, Basel, Bern, Zurich, and Lugano, as well as in the mountain HSA_O_s Sion and Davos.

The market share index is more difficult to interpret. High MSIs can be interpreted as an indication that HSA_O _residents prefer, or have no other choice than to be treated in their own HSA_O _hospitals. In contrast, low MSIs do not mean the opposite, but may indicate that the HSA_O _hospitals are also preferred for treatment by non-HSA_O _residents. Examples of HSA_O_s with a combination of a high LI (> 70%) and a low MSI (< 60%) are the HSA_O_s of the city (not the canton) of Bern, Einsiedeln, Sion, Davos, and St.Moritz. Also, high MSI may indicate that the HSA_O _hospitals are avoided (for whatever reason) by non-HSA_O _residents. In combination with low LIs, these HSA_O_s hospitals serve only a part of their own HSA_O _residents and not many non-HSA_O _residents. 21 (24.7%) such HSA_O_s (LI < 60% and MSI > 70%) can be observed. Whether such HSA_O_s provide suboptimal health care cannot be concluded from the data. Other factors such as differing regulations of hospitals, communes, regions, and cantons might play a role as well and should be analysed in detail. A third LI/MSI combination is comprised of HSA_O_s with high LI and MSI. With LI and MSI > 75%, Geneva, Lausanne, Montreux, Brig, and Lugano serve more of their own residents and fewer non- HSA_O _residents.

## Discussion

Small area analysis is a technique that uses large administrative data bases to study regional variation of healthcare resources and utilization. A persistent finding in health services research is that health care delivery and hospital utilization in the Western world vary widely between countries and between areas within countries [14,15]. This phenomenon is also observed in Switzerland. The reasons for geographic variations may be many – including the type of healthcare offered, socio-economic factors, and geographic influences – and have been discussed in detail by others [16-18]. Wennberg [18] concludes that the most plausible sources of variation in hospital utilization in small areas are organizational factors, including the distribution of resources, availability of beds, and balance between primary and secondary care. How relevant these sources of variation are to Switzerland cannot be determined from the descriptive data.

Although Switzerland's health care system is cantonally structured, patient streams may be influenced by multiple regulatory agreements among and between hospitals, communes, regions, and cantons. Discussions on the financing of hospitals currently are of great importance. For example, the "Neue Zürcher Zeitung" reported (2. Juni 2005) that the city of Zurich is demanding a reconfiguration of hospital regions because many non-city residents come to Zurich for treatment without their communities sharing the costs of health care. Small area analysis of hospital service areas can reveal new information and offer a new spatial model for hospital financing and planning.

The 3 introduced indices for patient streams allow the identification of areas with different characteristics -attractiveness, importance- of the provided hospital care and may guide health care planners in decision making processes. For example, although the method of creating HSAs implicates a tendency to regions with high LI, still HSAs may occur with LI < 50%. According to the method for building HSAs such a region has to be defined as a unique HSA, but still more than 50% of its residents are treated elsewhere in many different (surrounding?) HSAs. The implication of such a finding might be that the offered orthopedic supply in such an area should be questioned. Is it no longer needed or should the quality be revised? Furthermore, high positive netindices, not only draw the attention to HSAs with a centralized function (university or canton hospitals), but also -as in the case of Switzerland- to areas with special needs according to their geographic function (like regions within large ski areas). Finally, MSI in combination with LI can identify areas with "attractive" hospital(s) (high LI, low MSI) versus areas with "unattractive" hospital(s) (low LI, high MSI). From the patient's point of view the analyses show that healthcare availability itself (every HSA has at least one hospital) does not necessarily implement an equal attractive and/or comprehensive local supply of care.

### Limitations

This study has some limitations. Ideally, the definition of HSA_O_s should be based on a large number of observations collected over several years during which conditions or circumstances remain stable. However, the longer the period under study, the greater the possibility that some hospitals may be closed or newly opened. To a small degree this was observed in the data of 2000–2002: 9 HSA_O_s have more and 14 have fewer hospitals in 2002 than in 2000, which might influence observed patients flows. In addition, the data cannot discriminate hospital type (acute, rehabilitation, general, private, etc.). Rehabilitation clinics, for example, may attract many patients from outside their HSA_O_s and therefore should be excluded from the analysis when examining utilization of inpatient care. Furthermore, the underlying area model of medstat regions for building HSA_O_s was created using exclusively federal health statistics based on aggregated postal code areas; the aim was to achieve comparability of socio-demographic and geographic factors. How well such an aggregated area model represents the real world of patient streams remains unclear. Although postal codes might have yielded more precise estimates of the influence of patient utilization upon the size and shape of HSA_O_s, their use was precluded due to data confidentiality laws. Yet despite its aggregated nature, the medstat region may be considered as a reasonably valid area model for creating HSA_O_s. One last problem not considered in this paper is that HSA_O _definition should also include a criterion for minimum population size or number of discharges, because areas with a low population size may produce outlier values. However, it is unclear where to set a cut-off value, and the risk of combining HSA_O_s that do not belong together for geographic or demographic reasons might exist. The entire procedure of creating HSA_O_s would then become arbitrary and difficult to reproduce. Although individual HSA_O _values are treated as independent, in reality they are not; as Tobler's first law of geography states, "Everything is related to everything else, but near things are more related than distant things" [19]. HSA_O_s with central functions may automatically create low LI and negative netindices for their neighbouring HSA_O_s.

### Implications

Geographic information systems increasingly may allow communities, health care services researchers, and health care policy makers to link data sources, thus highlighting areas where public health interventions can be applied. The definition of health service areas for orthopedic procedures and the display of patient flows between such service areas can be used to characterize areas for their attractiveness for the local and nonlocal population. HSA_O_s may thereby reveal possible needs and demands which can help to assist hospital planning and policymaking.

Although these areas do not follow conventional municipal or cantonal borders, they may more closely represent the real world for hospital planning. Further investigations using overall discharge data for all procedures are on their way.

## Conclusion

Switzerland can be divided into 85 orthopedic hospital service areas. These areas show large variations with regard to patient streams and a trend to centralized health care services seen in the large urban areas, some central mountain areas, and isolated border regions. Older patients in general seem to be served more locally than children and adults; so too are patients who occupy a general bed. Large mountain sport area HSA_O_s serve more non-HSA_O _residents for emergencies than areas not located in the mountains. Patient-stream analyses on a small area level can help to identify areas with higher and lower attractiveness and importance for local and nonlocal residents, and may aid health care policy makers in hospital planning procedures.

## Competing interests

The authors declare that they have no competing interests. This project was supported by the National Research Program NRP 53 "Musculoskeletal Health – Chronic Pain" of the Swiss National Science Foundation (Project 405340-104607)

## Abbreviations

HSAo Orthopedic hospital service area

LI Localization index

MSI Market share index

## Authors' contributions

KM is responsible for drafting the manuscript. She defined the HSA_O_s, performed GIS operations, and calculated and analysed variables. AB carried out the statistics and contributed to the final version of the manuscript. All authors read and approved the final manuscript.

## Pre-publication history

The pre-publication history for this paper can be accessed here:



## Figures and Tables

**Table 1:** Mean values of overall orthopedic data per insurance class of the occupied beds, admission type or age group

	**Localization index** Median (min-max) Mean	**Netindex** %HSAo with a positive netindex	**Market share index** Median (min-max) Mean
**All data **n = 473217			

	60.8% (36.5–95.5%) 62.6%	30.6%	75.1% (39.1–94.9%) 72.2%

**Class of occupied bed**			

General Class n = 314416 (66%)	64.5% (41.9–96.2%) 65.2%^a,b^	31.8%	75.5% (42.8–95.3%) 73.3%^a^
Semi-private Class n = 82731 (17%)	53.6% (0–95.4%) 52.9%^a^	23.2%	71.4% (0–100%) 68.5%
Private Class n = 1680 (0.4%)	45.3% (14.4–94.2%) 47.9%^b^	27.0%	66.7% (25.7–93.7%) 65.7%^a^

**Admission type**^1)^			

Emergency procedures n = 128467 (27%)	74.3% (29.5–95%) 72.0%^a^	45.9%	76.9% (40.8–96.8%) 74.0%
Planned procedures n = 327541 (69%)	57.3% (21.2–95.9%) 58.0%^a^	25.9%	73.7% (35.5–95.0%) 72.7%

**Age group**			

<18 years old n = 33409 (7%)	56.0% (19.8–96.6%) 56.5%^a^	29.4%	72.4% (29.4–94.0%) 70.8%^a^
18–64 years old n = 285666 (60%)	55.4% (29.1–94.8%) 57.8%^b^	25.9%	72.0% (35.0–93.7%) 70.1%^b^
>64 years old n = 154142 (33%)	73.7% (47.1–92.2%) 72.9%^a,b^	41.2%	79.8% (44.7–97.9%) 76.8%^a,b^

^a,b^: significant values (p < 0.05) between dependent variables, 1) no data available for 2 HSA_o_
